# Uterine-cavity Lactobacillus relative abundance and endometrial polyp recurrence after hysteroscopic polypectomy: a retrospective cohort study

**DOI:** 10.3389/fpubh.2026.1958046

**Published:** 2026-10-07

**Authors:** Tingting Zheng, Cenchuan Guo, Zhan Zhou

**Affiliations:** 1 Department of Gynecology, Jinan Maternity and Child Care Hospital, Jinan, Shandong, China; 2 Department of Obstetrics and Gynecology, Huai’an Clinical Medical College of Jiangsu University (Huai’an Hospital of Huai’an City), Huai’an, Jiangsu, China; 3 Department of Gynaecology, Shandong Provincial Third Hospital, Jinan, Shandong, China

**Keywords:** 16S rRNA sequencing, endometrial polyp, hysteroscopic polypectomy, Lactobacillus relative abundance, recurrence, retrospective cohort, uterine microbiome

## Abstract

**Background:**

Endometrial polyps are among the most common benign uterine lesions, yet their recurrence after hysteroscopic polypectomy remains poorly understood. Emerging evidence suggests that uterine-cavity microbial composition may be related to endometrial inflammation and tissue biology. However, the association between uterine microbiome composition and polyp recurrence has not been systematically investigated.

**Methods:**

We conducted a single-center retrospective cohort study of 288 women who underwent hysteroscopic polypectomy between January 2021 and June 2024. Uterine cavity fluid was collected intraoperatively for 16S rRNA amplicon sequencing (V3–V4 region). Patients were classified into Lactobacillus-Dominant (LD, *n* = 156) and Lactobacillus-Non-Dominant (LND, *n* = 132) groups based on Lactobacillus relative abundance ≥70%. The primary outcome was endometrial polyp recurrence confirmed by hysteroscopy at 12 months. Multivariable Cox proportional hazards regression identified independent predictors of recurrence.

**Results:**

The 12-month recurrence rate was higher in the LND group than in the LD group (31.8% vs. 14.1%, *p* < 0.001). LND samples had higher relative abundances of Gardnerella (15.3%), Prevotella (12.1%), and Streptococcus (8.7%), higher Shannon diversity (3.87 ± 0.98 vs. 1.34 ± 0.52, *p* < 0.001), and higher serum IL-6 and CRP levels. In the multivariable Cox model, LND status remained associated with recurrence (adjusted HR 2.41; 95% CI 1.48–3.93; *p* = 0.001). BMI ≥ 28 kg/m^2^, polyp size ≥15 mm, multiplicity, and postoperative hormonal therapy were also associated with recurrence; the hormonal-therapy estimate indicated lower recurrence (adjusted HR 0.48). These estimates are observational and may be affected by residual confounding.

**Conclusion:**

Lactobacillus-non-dominant uterine-cavity microbiome status was associated with higher 12-month endometrial polyp recurrence after hysteroscopic polypectomy after adjustment for measured clinical factors. These findings support further evaluation of uterine microbiome profiles as potential risk-stratification markers, but they do not establish causality or justify routine antimicrobial or microbiome-guided treatment.

## 1 Introduction

Endometrial polyps are common benign focal lesions of the uterine cavity composed of endometrial glands, stroma, and a vascular core. They are frequently identified during evaluation of abnormal uterine bleeding, infertility, or incidental endometrial abnormalities, and hysteroscopic removal remains the standard approach when treatment is indicated (1–3). Although many patients improve after polypectomy, recurrence remains clinically relevant because recurrent lesions can lead to repeated bleeding, repeat hysteroscopy, additional pathology assessment, and renewed concern about fertility or endometrial disease. Recurrence after hysteroscopic polypectomy varies across studies and patient populations (4, 5).

Clinical factors associated with recurrence include previous polyp history, multiplicity, larger lesion size, and hormonal or metabolic characteristics (4). Postoperative hormonal management, including levonorgestrel-releasing intrauterine systems, has also been associated with lower recurrence in observational cohorts, although treatment selection is not randomized and therefore remains susceptible to confounding (5). These findings indicate that recurrence is unlikely to reflect a single pathway. Instead, it probably results from interactions among residual endometrial susceptibility, local tissue biology, hormonal stimulation, inflammation, and procedural factors. Identifying additional biological markers that remain associated with recurrence after adjustment for these clinical features could therefore improve mechanistic understanding and future risk stratification.

Interest in the endometrial and uterine microbiome has grown with the development of high-throughput 16S rRNA sequencing. Studies of infertility and implantation have challenged the historical assumption that the uterine cavity is uniformly sterile and have described low-biomass bacterial signals within endometrial samples (6). Work mapping microbial communities along the female reproductive tract further suggests that microbial composition changes across anatomical compartments and that uterine samples should not simply be treated as extensions of the vaginal microbiome (7). Lactobacillus-dominant endometrial profiles have been investigated in relation to reproductive outcomes, while non-Lactobacillus-dominant profiles have been associated with altered implantation environments in some cohorts (8). These observations provide biological context for studying uterine microbial composition in benign endometrial disease, while also emphasizing that findings from infertility populations cannot be directly extrapolated to polyp recurrence.

Evidence directly involving endometrial polyps is emerging. Barcoded sequencing has demonstrated diverse intrauterine bacterial profiles in women with endometrial polyps (9), and more recent 16S rRNA sequencing studies have reported differences in endometrial microbial composition between women with and without polyps (10). Reviews of uterine microbiome research likewise identify benign uterine disorders as a developing area of investigation but note substantial heterogeneity in sampling, sequencing, and analytical methods (11). Thus, the relevant literature is not absent, but most available studies are cross-sectional. Whether a microbiome profile measured around the time of polypectomy is associated with subsequent recurrence is substantially less well established.

This question is methodologically challenging because the uterine cavity is a low-biomass environment. Sampling route, contact with the cervix or vagina, reagent contamination, and laboratory background can influence detected microbial signals, and sampling technique itself can affect interpretation (12). General low-biomass microbiome guidance therefore emphasizes negative controls, contamination-aware bioinformatics, sequencing-batch assessment, and cautious interpretation of relative-abundance data (13, 14). These considerations are particularly important when a study classifies participants according to relative Lactobacillus abundance, because group definition can itself create expected differences in Lactobacillus proportion and in the relative proportions of other taxa. Accordingly, microbiome group comparisons should be interpreted as compositional associations rather than direct evidence of absolute bacterial overgrowth or microbial activity.

Inflammation provides one plausible, but unproven, link between microbial composition and polyp biology. Chronic endometrial inflammation has been described in association with several gynecologic conditions (15), and hysteroscopic, histologic, and bacteriologic studies have established chronic endometritis as a clinically recognizable inflammatory endometrial phenotype (16). Reviews further support a relationship between chronic endometritis and adverse reproductive or endometrial outcomes (17). Endometrial physiology is tightly regulated by inflammatory, vascular, stromal, and hormonal signals (18), while reproductive-tract microbiota can interact with host immune responses (19). Lactobacillus species also have recognized ecological and immunological roles in the lower reproductive tract (20). However, these observations do not prove that microbiome-associated serum cytokine differences mediate endometrial polyp recurrence.

Other uterine-disease studies provide additional context but also reinforce the need for cautious inference. Uterine microbiome alterations have been investigated in endometrial cancer (21), and endometrial microbial profiles have been characterized during embryo-transfer procedures (22). Chronic endometritis has also been associated with a higher risk of polyp recurrence after hysteroscopic polypectomy (23), supporting the broader possibility that inflammatory endometrial conditions may influence recurrence. At the same time, hysteroscopic removal itself is an effective treatment for symptomatic polyps (24), so completeness of initial resection, follow-up procedures, and postoperative management remain important alternative explanations for recurrent lesions. In addition, some asymptomatic endometrial polyps may regress spontaneously during follow-up (25), further indicating the heterogeneous natural history of endometrial polyps.

Against this background, a longitudinal assessment of uterine-cavity microbial composition at the time of polypectomy may help determine whether microbiome features are associated with later recurrence beyond measured clinical characteristics. The primary aim of this retrospective cohort study was therefore to determine whether Lactobacillus-non-dominant uterine microbiome status was associated, after adjustment for measured clinical factors, with hysteroscopically confirmed 12-month endometrial polyp recurrence after polypectomy. Secondary aims were to characterize group-level microbiome differences, examine Lactobacillus abundance as a continuous measure, evaluate exploratory associations with systemic inflammatory biomarkers, and assess whether the microbiome-recurrence association persisted after adjustment for major measured clinical confounders. Because the design is retrospective and inflammatory markers were measured in serum rather than endometrial tissue, all microbiome, inflammation, and recurrence findings were interpreted as associations rather than evidence of causal mediation.

## 2 Materials and methods

### 2.1 Study design and setting

This single-center retrospective cohort study was conducted at Jinan Maternity and Child Care Hospital, a tertiary gynecological center in Shandong, China, and included patients treated between January 2021 and June 2024. The current retrospective research project was approved by the Medical Ethics Committee of Jinan Maternity and Child Care Hospital on 12 August 2026 (Approval No. KY R-26-031) and was conducted in accordance with the Declaration of Helsinki. The 2026 approval pertains to the retrospective research use and analysis of previously collected clinical data and biological specimens; procedures governing the original clinical collection and storage of specimens followed institutional practice at the time of collection.

### 2.2 Study population

Eligible participants were women who underwent elective hysteroscopic polypectomy for one or more histologically confirmed endometrial polyps during the study period. Inclusion criteria: (1) age 18–65 years; (2) hysteroscopic polypectomy with intraoperative uterine cavity fluid collection for 16S rRNA sequencing; (3) histopathological confirmation of endometrial polyp; and (4) at least 12 months of post-procedural follow-up with hysteroscopic reassessment. Exclusion criteria: (1) antibiotic or probiotic use within 1 month prior to surgery; (2) concurrent endometrial hyperplasia or malignancy; (3) ongoing hormone replacement therapy (HRT) at baseline; (4) immunosuppressive therapy or autoimmune conditions; (5) incomplete 16S rRNA sequencing data (quality control failure); and (6) loss to follow-up before 12-month reassessment.

### 2.3 Microbiome sampling and 16S rRNA sequencing

Intraoperative uterine cavity fluid (0.5–1.0 mL) was aspirated using a sterile transcervical catheter before uterine irrigation. Samples were transferred to sterile cryogenic vials, frozen at −80 °C, and processed for 16S rRNA analysis. Genomic DNA was extracted using the MagPure Tissue & Blood DNA KF Kit (Magen, China). The V3–V4 hypervariable region was amplified using primers 341F/806R and sequenced on an Illumina MiSeq platform (paired-end 300 bp). Raw reads were processed using QIIME 2 (v2023.2), and taxonomic classification used SILVA v138. Samples with fewer than 10,000 high-quality reads were excluded. Because the uterine cavity is a low-biomass environment, contamination is an important concern. Available laboratory quality-control procedures were applied during processing; however, a complete set of extraction blanks, PCR-negative controls, environmental controls, and absolute bacterial-load measurements was not available for all cases. This is acknowledged as a methodological limitation, and relative-abundance findings are interpreted cautiously (10, 12).

Patients were classified as Lactobacillus-Dominant (LD, ≥70% relative abundance) or Lactobacillus-Non-Dominant (LND, <70%). The 70% threshold was used as a prespecified operational definition to facilitate group-based analysis; it should not be interpreted as a universally validated biological cutoff for a healthy uterine microbiome (6, 8). Continuous Lactobacillus relative abundance was therefore also examined as supportive evidence. Alpha diversity was assessed using the Shannon index and observed feature count, and beta diversity using Bray–Curtis dissimilarity with PERMANOVA. Accordingly, LND status is treated as an operational compositional category rather than a clinically validated diagnosis of uterine dysbiosis.

### 2.4 Outcome definitions

The primary outcome was confirmed endometrial polyp recurrence at 12 months post-polypectomy, defined as one or more new endometrial polyps ≥5 mm identified by office hysteroscopy at the scheduled 12-month follow-up assessment. Secondary outcomes included: recurrence at 6 months, time to recurrence (months from surgery to hysteroscopic confirmation), number of recurrent polyps, and the need for re-intervention. Serum inflammatory markers (IL-6, CRP, TNF-α) and serum estradiol were measured preoperatively as part of routine workup.

### 2.5 Statistical analysis

Baseline clinical characteristics were compared descriptively between LD and LND groups. Continuous variables summarized as mean ± SD were compared using two-sided independent-samples *t* tests, and categorical variables using Pearson chi-square tests; Fisher’s exact test was used when expected cell counts were small. The primary 12-month recurrence endpoint was summarized as a cumulative proportion. Because recurrence was assessed at discrete follow-up examinations, the true recurrence time is interval-censored. Conventional Kaplan–Meier and Cox analyses using the date of hysteroscopic confirmation as the event time were therefore considered exploratory and should not be interpreted as exact-time survival analyses. The multivariable Cox model included microbiome status together with measured clinically relevant covariates (BMI ≥ 28 kg/m^2^, polyp size, multiplicity, previous polyp history, and postoperative hormonal treatment). Lactobacillus relative abundance was additionally examined as a continuous exposure. Correlation analyses between microbiome indices and serum inflammatory biomarkers were exploratory and were not interpreted as mediation analyses. All tests were two-tailed; *p* < 0.05 was considered statistically significant. Analyses were performed using SPSS v27.0 and R v4.3.1.

## 3 Results

### 3.1 Patient flow and clinical characteristics

A total of 386 women underwent hysteroscopic polypectomy during the study period. After applying the eligibility criteria, 288 patients were included: 156 in the LD group and 132 in the LND group (Figure 1). The groups differed in several clinically relevant measured characteristics rather than being formally matched. The LND group had higher mean BMI (25.4 vs. 23.1 kg/m^2^, *p* < 0.001), a higher proportion with BMI ≥ 28 kg/m^2^ (28.8% vs. 14.1%, *p* = 0.002), larger mean polyp size (13.7 vs. 11.2 mm, *p* < 0.001), more multiple polyps (40.2% vs. 26.9%, *p* = 0.017), higher estradiol (172.6 vs. 148.3 pmol/L, *p* = 0.003), and higher inflammatory-marker levels. Postoperative hormonal therapy was less frequently used in the LND group (16.7% vs. 38.5%, *p* < 0.001) and is treated as a post-baseline exposure rather than a baseline characteristic (Table 1). These measured group differences were considered potential confounders in interpretation of the adjusted associations.

**Figure 1:**
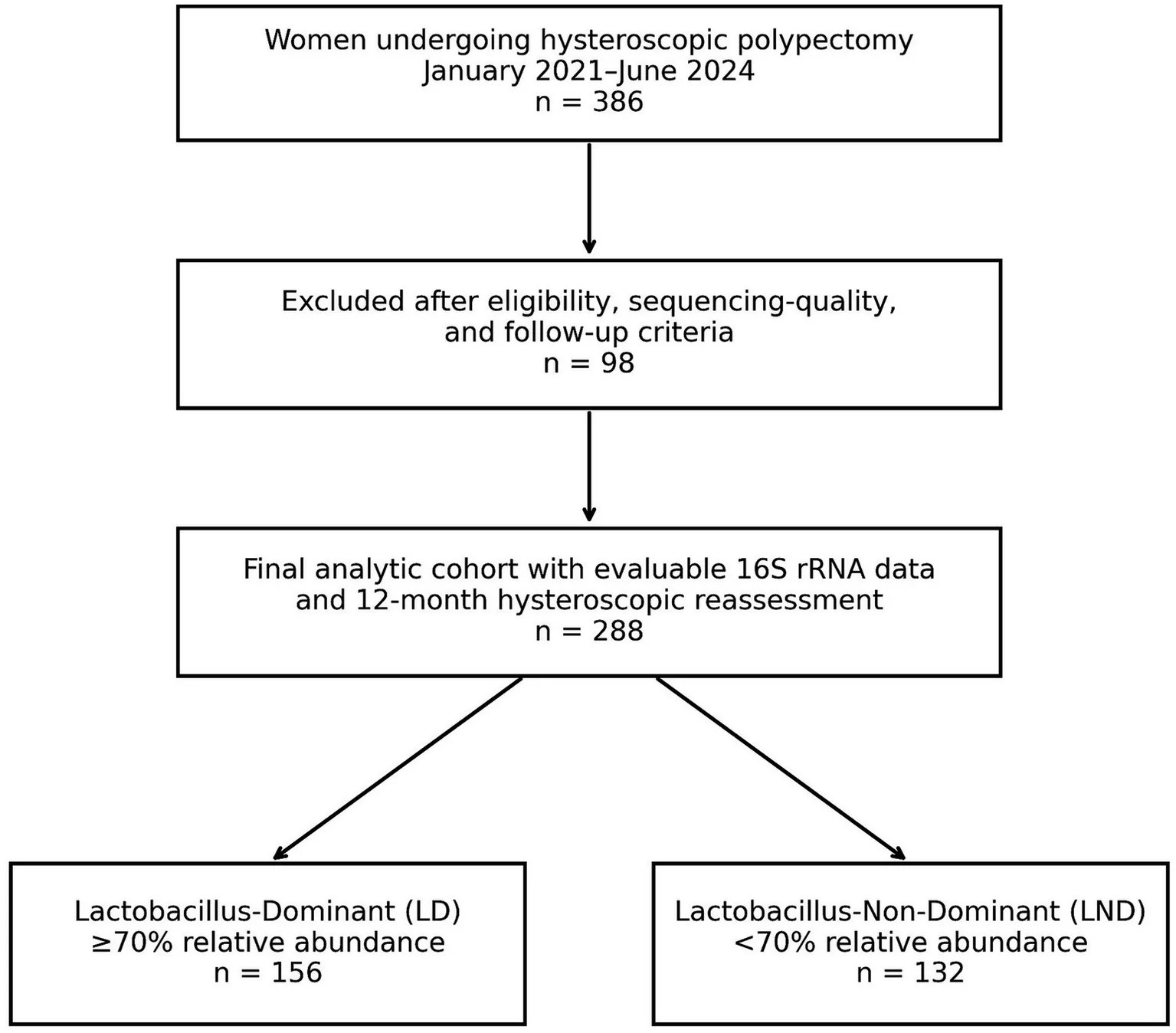
Study flow diagram depicting patient screening, eligibility assessment, microbiome classification, and inclusion in primary analysis.

**Table 1:** Clinical characteristics and postoperative hormonal treatment by uterine microbiome group.

Variable	LD Group (*n* = 156)	LND Group (*n* = 132)	*p*-value
Demographics			
Age (years), mean ± SD	42.3 ± 8.7	43.1 ± 9.2	0.450
BMI (kg/m^2^), mean ± SD	23.1 ± 3.2	25.4 ± 4.1	<0.001
BMI ≥ 28 kg/m^2^, *n* (%)	22 (14.1%)	38 (28.8%)	0.002
Parity ≥ 1, *n* (%)	107 (68.6%)	94 (71.2%)	0.629
Premenopausal status, *n* (%)	113 (72.4%)	86 (65.2%)	0.183
Polyp characteristics			
Polyp size, *n* (%)			0.030
<10 mm	64 (41.0%)	38 (28.8%)	
10–15 mm	60 (38.5%)	51 (38.6%)	
>15 mm	32 (20.5%)	43 (32.6%)	
Multiple polyps, *n* (%)	42 (26.9%)	53 (40.2%)	0.017
Previous polyp history, *n* (%)	33 (21.2%)	38 (28.8%)	0.134
Polyp size (mm), mean ± SD	11.2 ± 4.8	13.7 ± 5.6	<0.001
Comorbidities			
Hypertension, *n* (%)	29 (18.6%)	30 (22.7%)	0.386
Diabetes mellitus, *n* (%)	15 (9.6%)	19 (14.4%)	0.210
Postoperative hormonal therapy (OCP/LNG-IUD), *n* (%)	60 (38.5%)	22 (16.7%)	<0.001
Inflammatory and hormonal markers			
Serum estradiol (pmol/L), mean ± SD	148.3 ± 62.4	172.6 ± 74.1	0.003
Serum IL-6 (pg/mL), mean ± SD	4.2 ± 2.1	8.7 ± 3.4	<0.001
CRP (mg/L), mean ± SD	1.8 ± 1.2	4.3 ± 2.6	<0.001
Serum TNF-α (pg/mL), mean ± SD	6.4 ± 2.8	12.3 ± 4.7	<0.001

LD, Lactobacillus-Dominant group; LND, Lactobacillus-Non-Dominant group; BMI, body mass index; OCP, oral contraceptive pill; LNG-IUD, levonorgestrel intrauterine device; IL-6, interleukin-6; CRP, C-reactive protein; TNF-α, tumor necrosis factor-alpha; SD, standard deviation. Continuous variables were compared using two-sided independent-samples *t* tests and categorical variables using Pearson chi-square or Fisher’s exact tests as appropriate. Displayed *p*-values are descriptive and do not establish group comparability.

### 3.2 Uterine microbiome characterization

16S rRNA sequencing characterized the uterine-cavity samples from all 288 included patients. By definition, the LD group had substantially higher Lactobacillus relative abundance (88.3 ± 8.4% vs. 24.6 ± 18.2%). Shannon diversity and observed feature richness were higher in the LND group, and the relative abundances of Gardnerella, Prevotella, Streptococcus, Ureaplasma, and Bifidobacterium were also higher (Table 2; Figures 2A and 3). Because the groups were defined using Lactobacillus relative abundance and 16S data are compositional, these group differences are partly linked to the exposure definition and should not be interpreted as evidence of greater absolute bacterial loads. Bray–Curtis community composition differed between groups by PERMANOVA (*p* < 0.001).

**Table 2:** Uterine microbiome characteristics by group.

Microbiome metric	LD group (*n* = 156)	LND group (*n* = 132)	*p*-value
Alpha-diversity indices			
Lactobacillus relative abundance (%), mean ± SD	88.3 ± 8.4	24.6 ± 18.2	<0.001
Shannon diversity index, mean ± SD	1.34 ± 0.52	3.87 ± 0.98	<0.001
Observed feature count, mean ± SD	28.4 ± 12.3	87.6 ± 31.4	<0.001
Chao1 richness estimator, mean ± SD	31.2 ± 14.1	96.3 ± 38.7	<0.001
Dominant taxa in LND group (relative abundance)			
Gardnerella spp.	2.1 ± 1.8%	15.3 ± 6.4%	<0.001
Prevotella spp.	1.8 ± 1.4%	12.1 ± 5.8%	<0.001
Streptococcus spp.	1.4 ± 1.2%	8.7 ± 4.3%	<0.001
Ureaplasma spp.	0.8 ± 0.7%	6.4 ± 3.1%	<0.001
Bifidobacterium spp.	0.6 ± 0.5%	4.2 ± 2.0%	<0.001
Beta-diversity (Bray–Curtis dissimilarity)			
Within-group dissimilarity, mean ± SD	0.21 ± 0.09	0.54 ± 0.18	Descriptive
PERMANOVA group difference	—	—	<0.001

LD, Lactobacillus-Dominant; LND, Lactobacillus-Non-Dominant; PERMANOVA, permutational multivariate analysis of variance. Values are mean ± SD unless otherwise specified. “Observed feature count” is used because the manuscript does not document a separate OTU-clustering step. Relative-abundance comparisons are compositional and partly reflect the LD/LND classification rule; LND should not be interpreted as a clinically validated diagnosis of dysbiosis.

**Figure 2:**
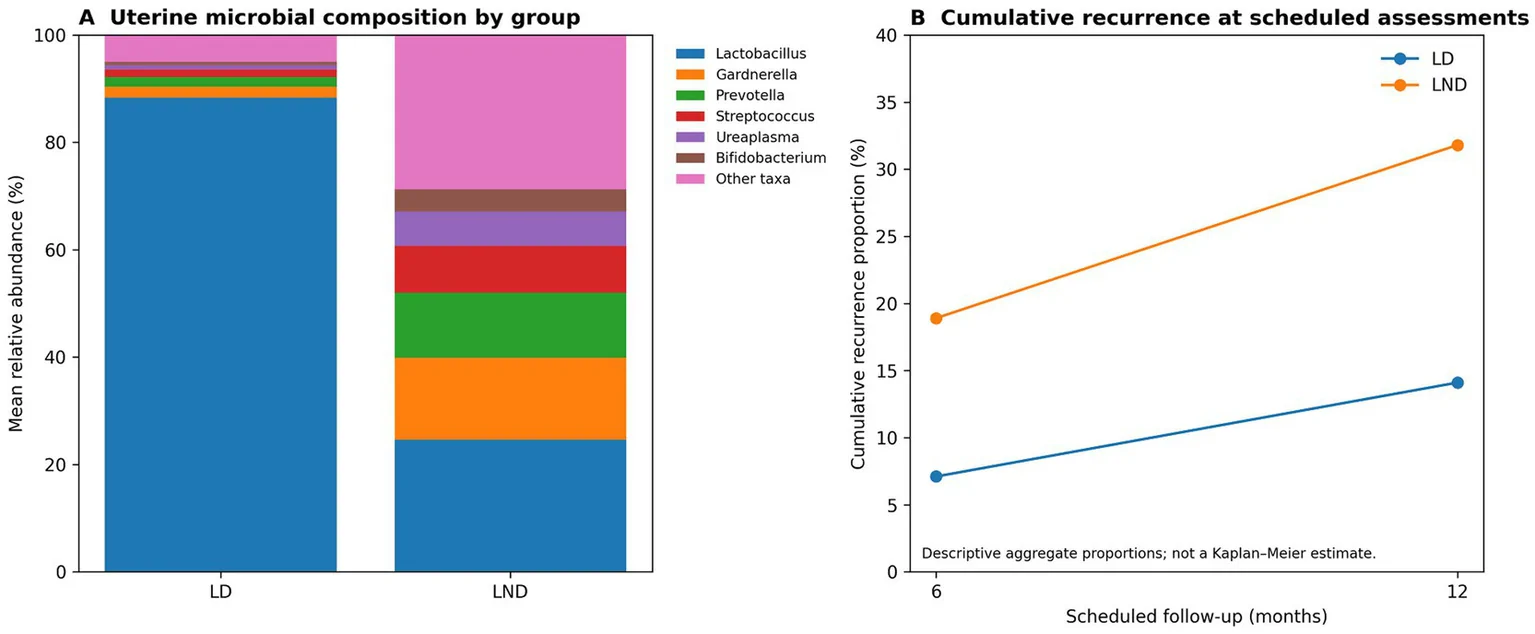
**(A)** Stacked bar chart of uterine microbial relative abundance by group. Because the groups were defined by Lactobacillus relative abundance, compositional differences should be interpreted cautiously. **(B)** Descriptive display of cumulative recurrence proportions at the scheduled 6- and 12-month assessments. This panel is not a patient-level Kaplan–Meier estimate and should not be interpreted as an exact-time survival curve.

**Figure 3:**
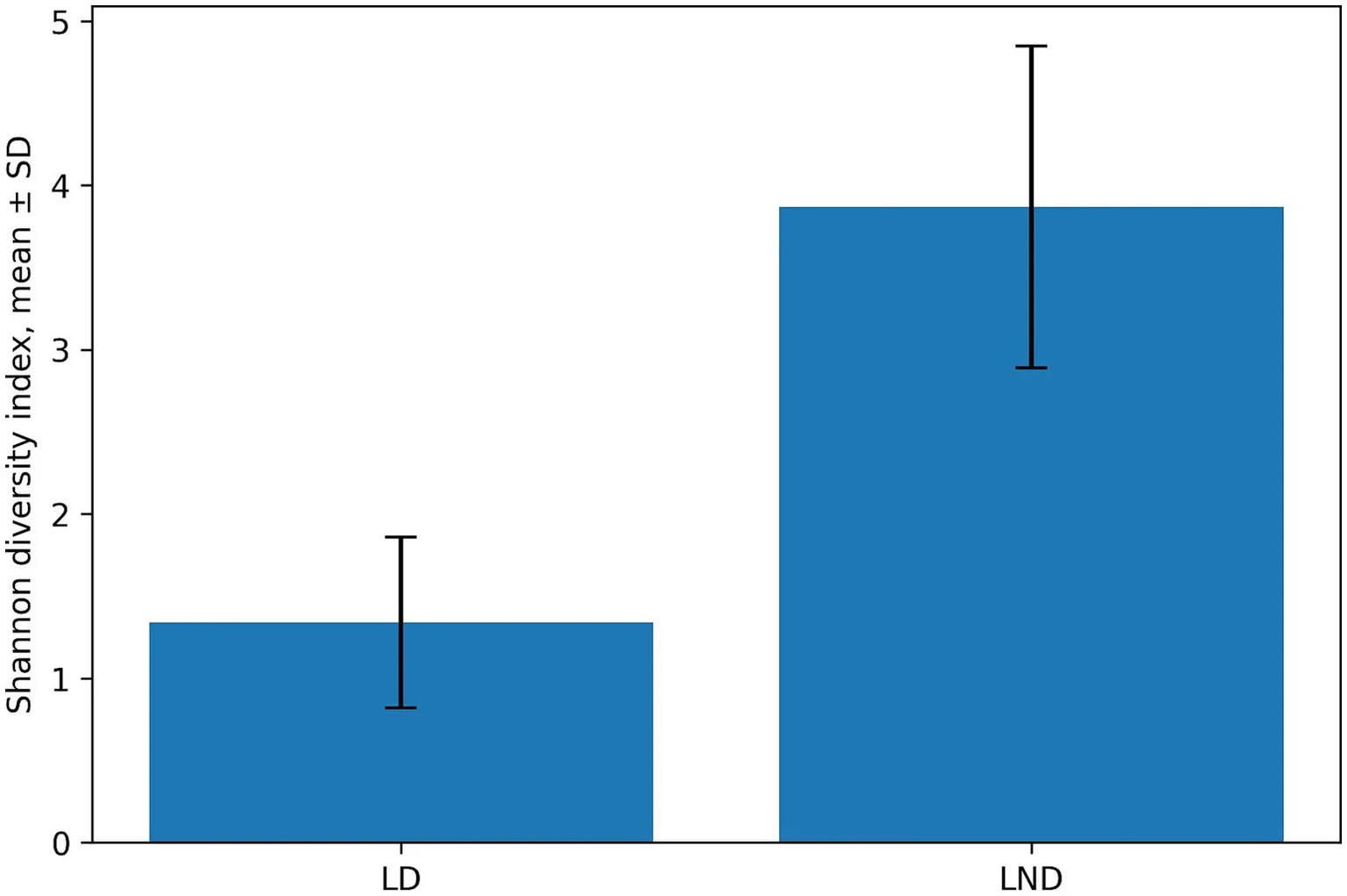
**(A)** Group summary of Shannon diversity by LD/LND status. **(B)** The association between Lactobacillus relative abundance and serum IL-6 should be displayed using participant-level measurements with an appropriate fitted line; a single-point display of the reported correlation coefficient is not interpretable as a scatterplot. Group summary of the Shannon diversity index by LD/LND status. Bars represent the mean ± SD.

### 3.3 Polyp recurrence at 12 months

The cumulative 12-month recurrence outcome occurred in 42/132 patients (31.8%) in the LND group and 22/156 (14.1%) in the LD group (Pearson chi-square *p* < 0.001). At the scheduled 6-month assessment, recurrence was recorded in 25/132 (18.9%) and 11/156 (7.1%), respectively (*p* = 0.002; Figure 2B). Because recurrence was detected at discrete follow-up assessments, the exact biological onset time was not observed. Accordingly, analyses that use the date of hysteroscopic detection as the event time are exploratory and subject to interval-censoring limitations. Full recurrence outcomes are presented in Table 3.

**Table 3:** Endometrial polyp recurrence outcomes at 6 and 12 months.

Outcome	LD group (*n* = 156)	LND group (*n* = 132)	*p*-value
Primary outcome			
Recurrence at 12 months, *n* (%)	22 (14.1%)	42 (31.8%)	<0.001
Secondary outcomes			
Recurrence at 6 months, *n* (%)	11 (7.1%)	25 (18.9%)	0.002
Median recurrence-free survival	Not reached	Not reached	—
Number of recurrent polyps among recurrent patients, mean ± SD	1.4 ± 0.6	2.1 ± 1.0	0.001
Recurrence requiring re-intervention, *n* (%)	14 (9.0%)	28 (21.2%)	0.003
Recurrence by polyp multiplicity			
Single polyp: recurrence rate	15/114 (13.2%)	24/79 (30.4%)	0.003
Multiple polyps: recurrence rate	7/42 (16.7%)	18/53 (34.0%)	0.057
Recurrence by postoperative hormonal treatment			
Treated (LNG-IUD/OCP)	5/60 (8.3%)	3/22 (13.6%)	0.441
Untreated	17/96 (17.7%)	39/110 (35.5%)	0.004

LD, Lactobacillus-Dominant; LND, Lactobacillus-Non-Dominant; IQR, interquartile range. Categorical comparisons use Pearson chi-square tests unless expected counts were small, in which case Fisher’s exact test was used. The number of recurrent polyps is summarized among patients with documented recurrence. The 12-month endpoint is a cumulative recurrence proportion. Time-to-event analyses are exploratory because recurrence was detected only at discrete follow-up assessments and exact recurrence times are interval-censored.

### 3.4 Adjusted associations with polyp recurrence

In the exploratory Cox analysis, LND status was associated with recurrence before and after adjustment for the measured covariates included in the model (adjusted HR 2.41; 95% CI 1.48–3.93; *p* = 0.001). BMI ≥ 28 kg/m^2^, polyp size ≥15 mm, multiple polyps, and postoperative hormonal therapy were also associated with recurrence (Table 4). The HR of 2.41 represents an estimated hazard-rate association and should not be interpreted as a 2.4-fold increase in cumulative risk. Because the groups differed in measured characteristics and potentially important confounders were unavailable, these estimates should be interpreted as adjusted associations rather than causal or validated predictive effects.

**Table 4:** Univariate and multivariable cox regression for 12-month polyp recurrence.

Variable	Univariate analysis			Multivariable analysis		
	Crude HR	95% CI	*p*	Adj. HR	95% CI	*p*
LND vs. LD (Lactobacillus-non-dominant vs. dominant)	2.58	1.60–4.17	<0.001	2.41	1.48–3.93	0.001
BMI ≥ 28 kg/m^2^ (vs. <28)	1.87	1.12–3.11	0.017	1.74	1.03–2.93	0.037
Polyp size ≥ 15 mm (vs. <15 mm)	2.12	1.28–3.52	0.004	1.96	1.17–3.29	0.011
Multiple polyps (vs. single)	1.94	1.19–3.17	0.008	1.82	1.10–3.01	0.020
Previous polyp history (yes vs. no)	1.68	0.98–2.87	0.059	1.54	0.89–2.66	0.122
Hormonal therapy post-op (yes vs. no)	0.52	0.28–0.97	0.040	0.48	0.25–0.92	0.028
Postmenopausal status (vs. premenopausal)	1.43	0.87–2.35	0.158	—	—	—
Age ≥ 50 years (vs. <50)	1.28	0.76–2.17	0.351	—	—	—
Diabetes mellitus (yes vs. no)	1.42	0.78–2.58	0.253	—	—	—

HR, hazard ratio; CI, confidence interval; Adj. HR, adjusted hazard ratio; LND, Lactobacillus-Non-Dominant; LD, Lactobacillus-Dominant; BMI, body mass index. The multivariable model included LND status, BMI ≥ 28 kg/m^2^, polyp size ≥ 15 mm, multiplicity, previous polyp history, and postoperative hormonal therapy. Schoenfeld residuals test: *p* = 0.624. With 64 recurrence events and six fitted covariates, the model provides approximately 10.7 events per fitted covariate and remains exploratory. Residual confounding cannot be excluded.

### 3.5 Subgroup analyses

Exploratory subgroup results are presented in Table 5. Point estimates were generally above 1.0, but confidence intervals were wide in several strata and no interaction test was statistically significant. These analyses were underpowered for effect-modification testing and should not be interpreted as demonstrating equivalence or broad applicability across subgroups.

**Table 5:** Subgroup analysis: association between LND and 12-month recurrence.

Subgroup	LD recurrence	LND recurrence	HR (95% CI)	Interaction *p*
By polyp size				
<15 mm	12/124 (9.7%)	21/89 (23.6%)	2.18 (1.08–4.40)	0.614
≥15 mm	10/32 (31.3%)	21/43 (48.8%)	1.82 (0.86–3.84)	
By BMI				
BMI < 28	16/134 (11.9%)	28/94 (29.8%)	2.37 (1.31–4.29)	0.358
BMI ≥ 28	6/22 (27.3%)	14/38 (36.8%)	1.54 (0.60–3.98)	
By polyp multiplicity				
Single polyp	15/114 (13.2%)	24/79 (30.4%)	2.28 (1.22–4.25)	0.812
Multiple polyps	7/42 (16.7%)	18/53 (34.0%)	2.02 (0.85–4.79)	
By postoperative hormonal treatment				
Treated	5/60 (8.3%)	3/22 (13.6%)	1.71 (0.40–7.28)	0.441
Untreated	17/96 (17.7%)	39/110 (35.5%)	2.50 (1.43–4.37)	

LD, Lactobacillus-Dominant; LND, Lactobacillus-Non-Dominant; HR, hazard ratio; CI, confidence interval; BMI, body mass index. Subgroup estimates are exploratory. Interaction *p*-values are from Wald tests; nonsignificant interaction tests do not establish equivalence between strata.

### 3.6 Microbiome-inflammation correlations

The LND group had higher serum IL-6 (8.7 ± 3.4 vs. 4.2 ± 2.1 pg./mL, *p* < 0.001), CRP (4.3 ± 2.6 vs. 1.8 ± 1.2 mg/L, *p* < 0.001), and TNF-α (12.3 ± 4.7 vs. 6.4 ± 2.8 pg./mL, *p* < 0.001). Shannon diversity correlated positively with IL-6 (*r* = 0.42), CRP (*r* = 0.38), and TNF-α (*r* = 0.35), whereas Lactobacillus relative abundance correlated inversely with IL-6 (*r* = −0.47) and CRP (*r* = −0.41) and positively with estradiol (*r* = 0.31); all reported correlation *p*-values were <0.001. In a separate exploratory logistic model, each 10-percentage-point increase in Lactobacillus relative abundance was associated with lower odds of 12-month recurrence (OR 0.78; 95% CI 0.68–0.89; *p* < 0.001). These analyses are associative and do not establish mediation or causality.

## 4 Discussion

In this retrospective cohort of 288 women undergoing hysteroscopic polypectomy, LND uterine microbiome status was associated with higher 12-month endometrial polyp recurrence after adjustment for measured clinical factors. The findings support a potential relationship between uterine microbial composition and recurrence, but the observational design does not establish causality or validate a clinical prediction model.

The observed associations with serum IL-6, CRP, and TNF-α provide a possible inflammatory context but should not be interpreted as evidence that inflammation mediates the microbiome-recurrence relationship. Cytokines were measured in serum rather than endometrial tissue or uterine fluid, and no formal mediation analysis or direct measurement of NF-κB, VEGF, COX-2, or matrix metalloproteinase activity was performed. Therefore, microbiome-inflammation mechanisms remain biologically plausible hypotheses rather than demonstrated pathways.

Lactobacillus species have recognized roles in reproductive-tract microbial ecology, but findings from the vaginal microenvironment cannot be directly extrapolated to the uterine cavity (19, 20). In the present cohort, Lactobacillus relative abundance was positively correlated with serum estradiol (*r* = 0.31), consistent with the value reported in Table 6. This association is exploratory and does not establish the direction or mechanism of a microbiome-hormone relationship.

**Table 6:** Exploratory associations between microbiome measures, biomarkers, and 12-month recurrence.

Correlation pair	Statistical test	Effect estimate	*p*-value
Diversity vs. inflammatory markers			
Shannon index × serum IL-6	Pearson r	*r* = 0.42	<0.001
Shannon index × serum CRP	Pearson r	*r* = 0.38	<0.001
Shannon index × serum TNF-α	Pearson r	*r* = 0.35	<0.001
Observed feature count × serum IL-6	Pearson r	*r* = 0.39	<0.001
Lactobacillus abundance vs. biomarkers			
Lactobacillus abundance × serum IL-6	Pearson r	*r* = −0.47	<0.001
Lactobacillus abundance × serum CRP	Pearson r	*r* = −0.41	<0.001
Lactobacillus abundance × serum estradiol	Pearson r	*r* = 0.31	<0.001
Lactobacillus abundance × 12-month recurrence	Logistic regression (OR per 10% ↑)	OR = 0.78 (0.68–0.89)	<0.001
Group differences in inflammatory markers			
Serum IL-6 (pg/mL): LD vs. LND (mean ± SD)	Independent *t*-test	4.2 ± 2.1 vs. 8.7 ± 3.4	<0.001
Serum CRP (mg/L): LD vs. LND (mean ± SD)	Independent *t*-test	1.8 ± 1.2 vs. 4.3 ± 2.6	<0.001
Serum TNF-α (pg/mL): LD vs. LND (mean ± SD)	Independent *t*-test	6.4 ± 2.8 vs. 12.3 ± 4.7	<0.001

LD, Lactobacillus-Dominant; LND, Lactobacillus-Non-Dominant; IL-6, interleukin-6; CRP, C-reactive protein; TNF-α, tumor necrosis factor-alpha; OR, odds ratio per 10-percentage-point increment in Lactobacillus relative abundance. Pearson correlations, logistic-regression estimates, and between-group comparisons represent distinct exploratory analyses and should be interpreted separately. None establishes mediation or causality.

Postoperative hormonal treatment (LNG-IUD or OCP) was associated with lower recurrence in the adjusted model (adjusted HR 0.48). Because treatment was not randomized and differed substantially between microbiome groups, this estimate should be interpreted as an association rather than a causal protective effect. Prior observational evidence suggests that LNG-IUS use after hysteroscopic polypectomy may be associated with lower recurrence (5). Further prospective studies are needed to clarify treatment effects and potential interactions with microbiome status.

BMI ≥ 28 kg/m^2^ was associated with recurrence in the adjusted model (adjusted HR 1.74). Adiposity is biologically relevant to endometrial hormonal and inflammatory physiology, but the present study does not establish a mechanistic pathway. The higher BMI and obesity prevalence in the LND group further emphasize the possibility of residual confounding between metabolic characteristics, microbiome composition, and recurrence.

Several limitations should be acknowledged. First, the retrospective design introduces selection bias and precludes causal inference. Second, the uterine cavity is a low-biomass environment, and contamination from reagents or the lower genital tract cannot be completely excluded; complete negative-control and absolute bacterial-load data were not available for all samples (12–14). Third, single-timepoint sequencing does not capture longitudinal microbiome dynamics. Fourth, the 70% LD/LND threshold is an operational cutoff and is not a universally validated threshold for uterine health; therefore, the LND category should not be interpreted as definitive dysbiosis, and continuous Lactobacillus analyses provide important supporting context. Fifth, LD and LND groups differed in BMI, polyp characteristics, hormonal treatment, estradiol, and inflammatory markers. Multivariable adjustment reduces but cannot eliminate residual confounding. Sixth, recurrence was assessed at discrete follow-up examinations, so exact recurrence times are interval-censored and conventional Kaplan–Meier/Cox analyses based on detection dates should be viewed as exploratory approximations rather than exact-time survival analyses. Finally, the single-center design may limit generalizability. Prospective studies with standardized negative controls, absolute bacterial-load measurements, serial sampling, appropriate compositional methods, and external validation are needed.

## 5 Conclusion

Lactobacillus-non-dominant uterine microbiome status was associated with higher 12-month endometrial polyp recurrence after hysteroscopic polypectomy after adjustment for measured clinical factors. This finding should be interpreted as an observational association rather than evidence of a causal microbiome-mediated inflammatory mechanism. Uterine microbiome profiling remains investigational, and the present data do not support routine antimicrobial prophylaxis or routine microbiome-guided management. Prospective studies with rigorous low-biomass controls and external validation are warranted.

## Data Availability

The datasets presented in this study can be found in online repositories. The names of the repository/repositories and accession number(s) can be found in the article/Supplementary Material.
